# Indirect DNA extraction method suitable for acidic soil with high clay content

**DOI:** 10.1016/j.mex.2018.02.005

**Published:** 2018-02-22

**Authors:** Eva Högfors-Rönnholm, Stephan Christel, Sten Engblom, Mark Dopson

**Affiliations:** a Research and Development, Novia University of Applied Sciences, Vaasa, Finland; b Centre for Ecology and Evolution in Microbial Model Systems (EEMiS), Linnaeus University, Kalmar, Sweden

**Keywords:** Indirect DNA extraction, Microbial community, Extracellular DNA, Acid sulfate soil, 16S rRNA gene

## Abstract

DNA extraction is an essential procedure when investigating microbial communities in environmental samples by sequencing technologies. High clay soils can be problematic as DNA adsorbs to the clay particles and can thereby be preserved from lysed, non-viable cells for a substantial period of time. In order to accurately estimate the intact and living microbial community in the soil, extracellular DNA from dead, remnant bacterial cells needs to be removed prior to DNA extraction. One possibility is to use a sodium phosphate buffer to release both extracellular DNA and bacterial cells from the clay particles. After removing the extracellular DNA by centrifugation, the remaining viable cells can be harvested and DNA extracted. The described method is a modification of a procedure for separating extracellular DNA and bacterial cells from acidic clay soils.

• The modified method increases bacterial cell yields from acidic clay soils, such as acid sulfate soil.
• The modified method eliminates some steps from the original method, as only DNA from intact bacterial cells is required.
• The indirect DNA extraction method increases the workload compared to standard direct extraction methods, but subsequent downstream analyses will give a more representative picture of the viable microbial community composition in the soil.

## Graphical abstract

**Figure:**
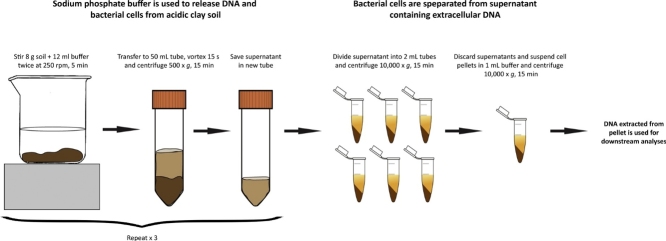

## Method details

Co-extraction of both intracellular DNA from living bacterial cells and extracellular DNA (eDNA) from dead cells by using standard direct DNA extraction methods can result in a considerable overestimation of the current, living microbial community in soil [1]. When working with clay rich soils, it is important to take into consideration that clay particles are known to preserve extracellular gene sequences due to their strong adsorptive binding of nucleic acids [2] as well as nucleases [3]. Therefore, if you do not separate the intact bacterial cells from the eDNA bound to the clay particles prior to DNA extraction, the subsequent high-throughput DNA sequencing will not be able to discriminate current and living bacterial species from remnant species no longer inhabiting the soil. This will thereby result in a skewed picture of the soil biodiversity [1]. In this paper, a modified indirect DNA extraction method based on a procedure for the separate recovery of intra- and extracellular DNA from marine samples by Alawi et al. [4] is described. The method uses a sodium phosphate buffer for the simultaneous release of both eDNA and bacterial cells from the soil. The method has further been optimized for acidic clay soil by adjusting the soil to buffer ratio, improving the separation procedure, and increasing the sodium phosphate buffer concentration [5] to increase cell yields. Only separated, intact bacterial cells are used for the subsequent DNA extraction and thereby, downstream analyses will only take into consideration the current, living microbial community.

### Reagents

Sodium phosphate buffer (500 mM Na_2_HPO_4_ and 500 mM NaH_2_PO_4_, pH 7.2)

### Indirect DNA extraction protocol

1. 8 g of homogenized fresh clay soil is weighed into a sterile 100 mL beaker, containing a sterile magnetic stirring bar and covered with aluminum foil.
2. 12 mL of sodium phosphate buffer (soil to buffer ratio 1:1.5) is added to the beaker.
3. The slurry is stirred for 5 min at 250 rpm.
4. The beaker is chilled for 3 min at 4 °C.
5. The slurry is stirred again for 5 min at 250 rpm.
6. The slurry is transferred to a 50 mL sterile tube and mixed by vortex for 15 s.
7. The tube is centrifuged at 500*g* for 15 min.
8. The supernatant is carefully poured into a separate 50 mL sterile tube and stored in the dark at room temperature.
9. 12 mL of fresh sodium phosphate buffer is added to the pellet and the pellet is suspended by careful repeated pipetting using a 5 mL pipet. The slurry is transferred back to the beaker and the procedure described above (steps 3–8) is repeated a further two times.
10. All supernatants are collected in the same 50 mL tube and the final soil pellet is discarded.
11. The pooled supernatant (approximately 30 mL) is divided into sterile 2.0 mL tubes and centrifuged at 10,000*g* for 15 min to separate the cells from the suspension. Extracellular DNA is left in the supernatant.
12. The supernatants are discarded and the cell pellets washed by pooling them into a 2.0 mL tube with 1 mL sodium phosphate buffer to remove excess eDNA.
13. The tube is centrifuged at 10,000*g* for 15 min and the supernatant is discarded.
14. If DNA is extracted using a commercial DNA isolation kit, the cell pellet is transferred to the kit́s bead tube using the bead tube buffer and the DNA is extracted according to the manufactureŕs instructions.
15. The indirectly extracted DNA can now be used for suitable downstream analyses.

### Notes on the protocol

1. The autoclaved sodium phosphate buffer is best stored in room temperature as it quickly crystallizes in lower temperatures.
2. If soil samples with higher salt concentrations are used, 2% NaCl can be added to the sodium phosphate buffer to reduce the osmotic stress on the bacterial cells.
3. Sometimes the final cell pellet after the washing step (points 12–13 in the protocol) can be hard and difficult to suspend in the bead tube buffer (point 14 in the protocol). To avoid losing cell material, pool the cell pellets in up to four tubes/sample instead of one tube to reduce the pellet amount per tube.
4. The used commercial DNA isolation kit was the DNeasy Power Soil Kit (Qiagen).

### Method validation

**Fig. 1:**
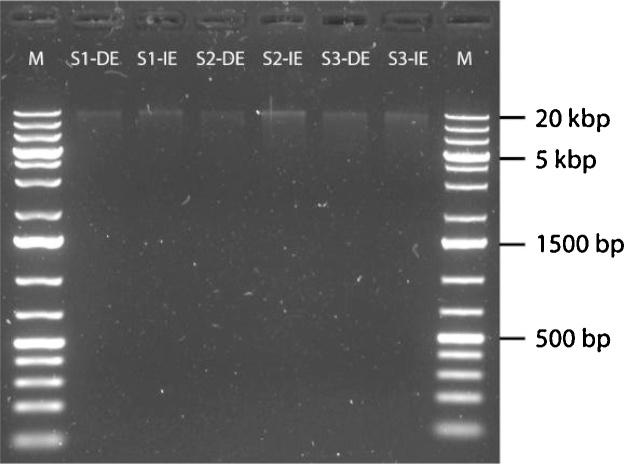
Gel electrophoresis (1% agarose) of DNA extracted directly from soil (DE) and DNA extracted indirectly from soil using the indirect DNA extraction protocol for acidic soil with high clay content (IE). Three biological replicate acid sulfate soil samples taken several meters apart (S1, S2 and S3) were used. Lane M: GeneRuler 1 kb Plus DNA Ladder (Thermo Scientific).

**Fig. 2:**
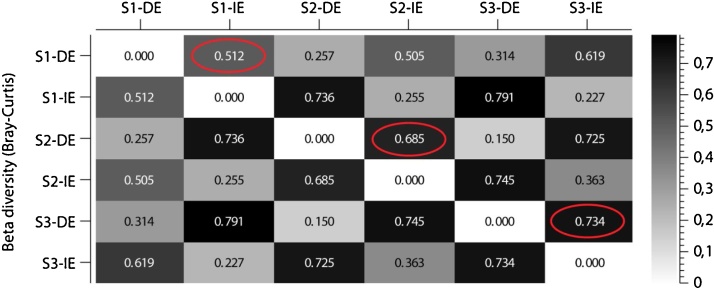
Bray-Curtis beta diversity analysis on bacterial community between DNA extracted directly from soil (DE) and DNA extracted indirectly from soil using the indirect DNA extraction protocol for acidic soil with high clay content (IE). Three biological replicate acid sulfate soil samples taken several meters apart (S1, S2 and S3) were used. The red circles point out the bacterial community dissimilarity indexes in corresponding soil samples.

**Fig. 3:**
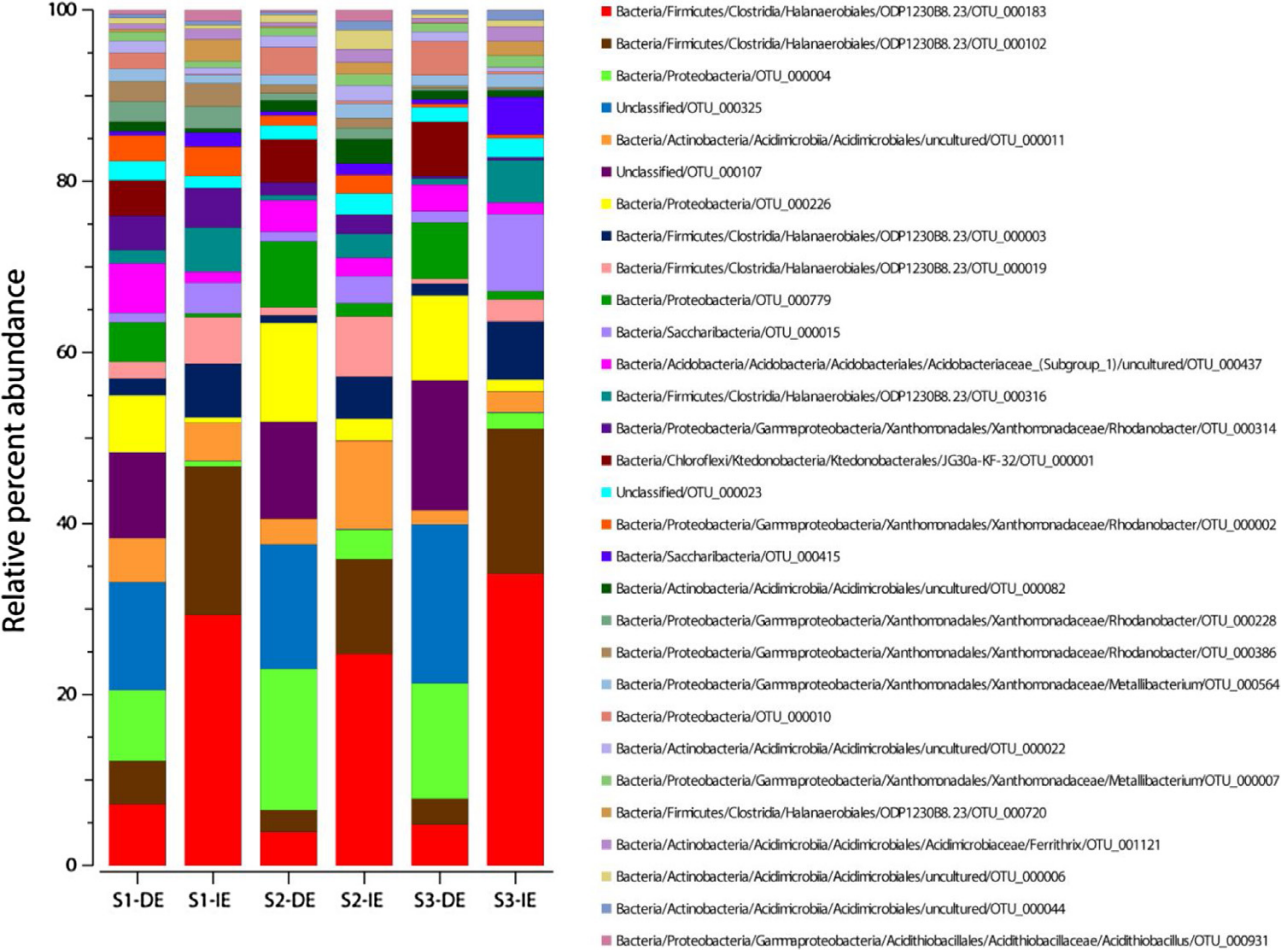
Stackbar graphs with the relative percent abundances of the top 30 OTUs in the 16S rRNA gene sequencing of DNA extracted directly from soil using a DNA extraction kit (DE) and DNA extracted indirectly from soil using the indirect DNA extraction protocol for acidic soil with high clay content (IE). Three biological replicate acid sulfate soil samples taken several meters apart (S1, S2 and S3) were used.

The indirect DNA extraction protocol has successfully been used to describe the intact microbial community in clay rich acid sulfate soil [6]. The indirect method described above followed by the DNeasy PowerSoil Kit produced 2.23 ± 0.21 ng DNA/μL (*A*_260/280_ = 1.97–2.09 and *A*_260/230_ = 0.79–1.18) while the direct extraction using solely the DNeasy PowerSoil Kit produced 1.41 ± 0.36 ng DNA/μL (*A*_260/280_ = 1.73–2.49 and *A*_260/230_ = 0.43–1.11). Purity of extracted DNA (Fig. 1) did not interfere with PCR amplifications (data not shown) or sequencing. High throughput 16S rRNA gene sequencing on the Illumina platform [6] of DNA extracted directly from the soil (0.26–0.37 million reads) compared to the indirect method (0.30–0.38 million reads) showed a dissimilarity in microbial community composition in three biological replicate soil samples (Bray-Curtis community dissimilarity index calculation; Fig. 2). Furthermore, the top 30 operational taxonomic units (OTUs) identified in three biological replicate samples also showed the dissimilarity in bacterial community between direct and indirect DNA extraction methods (Fig. 3). Even when looking at the most abundant OTUs in the samples, one can see a shift in bacterial community structure depending on the DNA extraction method used. The direct extractions in Fig. 3 shows three OTUs (OTU_00001, OTU_000107, and OTU_000325) that are missing in the indirect extraction and indicates that these OTUs were only present as eDNA from remnant species. On the other hand, one can see that the current intact bacterial community in the indirect extractions is dominated by species from order Halanaerobiales and this observation would have been overlooked if only a standard direct extraction method had been used. The relative percent abundance of species most similar to known acidophiles such as *Acidithiobacillus* sp. (OTU_000931) and *Ferrithrix* sp. (OTU_001121) is furthermore higher in indirect extracted samples, which is logical since the samples were taken from an active acid sulfate soil. More details about the validation of the method can be found in the publication [6] and its supplemental files. Based on these validations, we concluded that this indirect DNA extraction method was more representative for the description of the microbial community in acidic soil with high clay content, but can moreover suit other problematic soils preserving eDNA. In addition to the procedure our method is based on [4], other authors have proposed several indirect DNA extraction methods suitable for soil samples (see e.g. ref [7]) as well as other methods using propidium monoazide to differentiate eDNA from intracellular DNA of intact, living cells [8]. However, these methods are not suitable for (acidic) clay soil or are too chemical or mechanical damaging on the bacterial cells adhered to the clay particles.
